# Calvarial lesions: overview of imaging features and neurosurgical management

**DOI:** 10.1007/s10143-021-01521-5

**Published:** 2021-03-22

**Authors:** Isabella Nasi-Kordhishti, Johann-Martin Hempel, Florian Heinrich Ebner, Marcos Tatagiba

**Affiliations:** 1 Department of Neurosurgery, Eberhard Karls University Tübingen, Hoppe-Seyler-Strasse 3, 72076 Tübingen, Germany; 2 Department of Neuroradiology, Eberhard Karls University Tübingen, Tübingen, Germany; 3 Department of Neurosurgery, Alfried Krupp Hospital, Essen, Germany

**Keywords:** Calvarial lesions, Imaging, Histiocytosis, Meningiomas, Metastases

## Abstract

Calvarial lesions are rare and can present as a variety of different diseases. The lesions can be palpable on the skin and cause local pain and paraesthesia and, depending on the location, neurological deficits can also occur. This research aims to present an overview of typical imaging features as well as neurosurgical management. We examined the charts of patients who underwent surgery on a calvarial lesion in our department between 2004 and 2017 (*n*=133). Retrospectively, the pre-, intra-, and postoperative data were analyzed with morphological and histological findings and compared with each other. Pain, swelling, cosmetically disturbing, and neurological deficits were the main complaints. Seventy-seven lesions were limited to the bone, while another 56 lesions showed an infiltrating growth in the adjacent tissue. Depending on the clinical signs and suspected diagnosis, a biopsy, a partial removal, or a complete resection was performed. Histiocytosis (*n*=20), meningiomas (*n*=20), metastases (*n*=19), and osteomas (*n*=16) were the most common lesions. Fibrous dysplasia (*n*=6) and intraosseous hemangioma (*n*=9) were less common; other lesions were present only in isolated cases. Imaging features may suggest the lesion to be benign or malignant, but the diagnosis can be only confirmed by histological examination. The surgical strategy depends on the complaints, location of the lesion, and suspected diagnosis. Adjuvant treatment should be initiated according to the histological findings.

## Introduction

Intraosseous lesions of the calvarium are often slow, progressive processes that manifested in swelling, local pain, or sensitivity disorders. Asymptomatic processes are often described as random findings in the imaging. A variety of diseases can hide behind such a lesion. We distinguish between lesions that originate primarily from the bone and those that present a secondary bone infiltration. Depending on the author, the first category is divided into three or four subcategories: primary neoplasia (in individual papers already divided into good and malignant primary neoplasia), secondary neoplasia, and tumor-like lesions [17, 22]. Using adequate imaging as computed tomography (CT) and magnetic resonance imaging (MRI), suspicions can be drawn about the dignity of the present lesion, especially if the clinical symptoms and the case history of the patients are included [8, 12, 14]. A definitive diagnosis of a lesion can be only made based on histopathological examination. The clinical management methods of calvarial lesions differ greatly depending on the underlying entity [17]. Most benign lesions, such as osteomas, meningiomas, or intraosseous hemangiomas, can usually be completely resected and do not need further treatment [5, 16, 18, 23]. However, malignant lesions, such as metastasis or osteolysis in plasmacytoma, potentially require an adjuvant therapy such as chemotherapy, immunotherapy, or radiation [3, 17]. Rare diseases such as histiocytosis require individual diagnostic and therapeutic evaluations [2, 20].

Since calvarial lesions are rare diseases in neurosurgery, only a few single-case reports and reviews have been published. This research aims to present an overview of calvarial lesions based on typical imaging features and the histopathological results, as well as the neurosurgical management.

## Materials and methods

Included are all children and adults with lesion of the clavarium and orbita who were operated in our department between 2004 and 2017. The pre-, intra-, and postoperative data, as well as the imaging and histopathological findings, have been analyzed retrospectively. All patients with solitary lesions at the skull base were excluded. Since this is a surgical series, patients who have been treated only conservatively are not included. This case series does not claim to be a presentation of all possible disease entities.

All statistical tests were performed using JMP 13 (SAS Institute Inc., Cary, NC). Descriptive data are shown as mean, standard deviation (SD), and percentage.

## Results

### Pre- and intraoperative evaluation

**Fig. 1:**
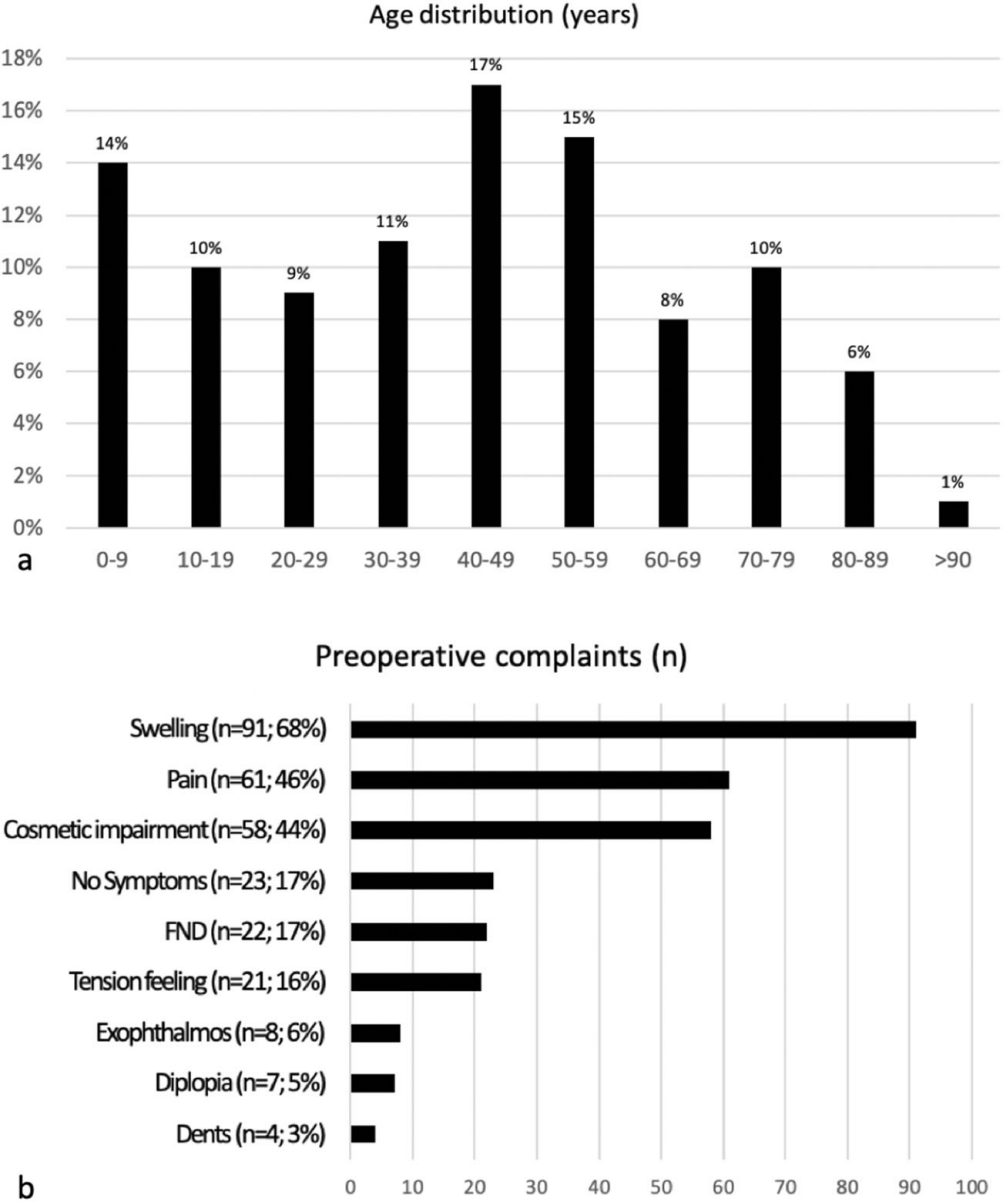
**a** Age distribution in years. **b** Preoperative complaints. FND, focal neurological deficits

A total of 133 patients were operated, 74 of whom were women, and 59 were men. The age distribution of 0–95 years is homogeneously distributed over all decades with a mean of 42 years and a standard deviation (SD) of 24.7 (Fig. 1a).

The patients’ main complaints (Fig. 1b) were swelling (*n*=91; 68%), pain (*n*=61; 46%), and subjective cosmetic impairment (*n*=58; 44%). Rarely represented were focal neurological deficits (FND) (*n*=22; 17%), feelings of tension of the skin (*n*=21; 16%), exophthalmos (*n*=8; 6%), diplopia (*n*=7; 5%), and dents in the skin (*n*=4; 3%). Only 23 patients have not complained of any symptoms.

The location of the lesions is distributed in every part of the skull with a clear frontal prevalence (*n*=55; 41%). Parietal (*n*=26; 19%), occipital (*n*=20; 15%), orbital (*n*=12; 9%), sutural (*n*=7; 5%), and temporal (*n*=4; 3%) lesions are rarer. In two newborns, a lesion was found directly on the fontanel (1.5%).

All patients, except the two newborns, have received preoperative cranial imaging using CT and MRI with and without contrast enhancement. The newborns were dispensed with CT diagnostics. Also, an X-ray, sonography, PET-CT, or bone scintigraphy was performed preoperatively in individual cases. The typical findings for the lesions are described in Table 1 after the classification of the postoperatively obtained histology.

**Table 1:** Imaging and intraoperative macroscopically findings of calvarial lesions after histological diagnosis. *CFD*, color flow doppler sonography; *CNS*, central nervous system; *CSF*, cerebrospinal fluid; *CT*, computed tomography; *MRI*, magnetic resonance imaging; *PET*, positron emission tomography

Tomography findings CT and MRI	Other imaging methods	Macroscopically intraoperative
Primary neoplasia		
Arachnoidal cyst (*n*=1; 0.75%)		
- Calvarial (Tabula interna and externa) thinning - Well-circumscribed cyst containing CSF (isodense, isointense)		- rachnoidal Bubbles - Stretched veins - No solid mass
Dermoid cyst (*n*=7; 5%)		
- Cystic lesion containing fat density - Fat in CT hypodense and in MRI hyperintense - In 20% calcification of the wall	Sonography: - Well-defined lobulated mass - Echo-rich/nonhomogeneous contents - Lack of ossification	- Circumscribed mass - Dents in the bone - Fat tissue - Bone destruction
Epidermal cyst (*n*=2; 1.5%)		
- Round, lobulated lesion - Heterogeneous signal - Possible restricted diffusion		- Lytic bone lesion filled with keratinous material - Liquid/gelatinous content
Epidermoid cyst (*n*=7; 5%)		
- Round, lobulated lesion - CT: sharply demarcated lytic lesion - MRI: T1 often slightly hyperintense to CSF T2 often iso-/slightly hyperintense to CSF - DWI: increased signal distinguishes cystic lesion from arachnoid cyst	Sonography: - Partial destruction of the tabula externa - CFD: no perfusion - Completely intraosseous cyst X-ray: - Lytic bone lesion	- Destroyed bone - Liquid, whitish pearl-colored mass - Vascularized skin - Bone thinning
Fibroma (*n*=1; 0.75%)		
- CT: sharply demarcated lytic lesion - MRI: no specific signal changes	Skeletal scintigraphy: - Tracer uptake	- Whitish, soft - Lytic bone lesion
Hyperostosis (*n*=9; 7%)		
- CT: osseous protrusion of the calvaria, starting from the tabula externa - MRI: no specific signal changes	Skeletal scintigraphy: - Focal uptake	- Only tabula externa affected
Intraosseous hemangioma (*n*=9; 7%) Fig. 2e		
- CT: lytic-cystic lesion confined to the bone with involvement of the tabula interna and externa. - Low density. - No dura infiltration - MRI: contrast enhancement. T2 possible flow voids in large vessels T2* possible susceptibility artifact due blood breakdown in the vessels		- Palpable swelling - Hypervascularized - Soft consistency - Bluish to purple-reddish color - Fibrotic capsule
Meningioma (*n*=20; 15%) Fig. 2a–d WHO I Fig. 2a–b		
- MRI: extra-axial mass with dural adhesion; homogeneous contrast enhancement; “dural tail” in 30–85% of cases, but unspecific; T2: flow voids, possibly “sunburst” phenomenon - CT: bone thickening; transosseous growth; irregular cortex; hyperostosis; often calcifications - Necrosis and cystic parts are common, hemorrhage is rarely	X-ray: - Callus formation	- Infiltrative growth - Exophytic - Bone surface changed and uneven - Tumor intraosseous hard, intracranially soft - Hypervascularized - Dura can not be clearly defined - Reddish color
WHO II		
- Like WHO I - Correlation between increased tumor grade and imaging with blurred tumor-brain delimitation, capsular, and heterogeneous enhancement	Skeletal scintigraphy: - No evidence of malignancy PET: - Metabolically active, osteodestructive	- Avascular to hypervascularized - Swelling above bone level - Infiltrating growth - Tough, reddish
WHO III Fig. 2c–d		
- Like WHO I - Correlation between increased tumor grade and imaging with blurred tumor-brain delimitation, capsular and heterogeneous enhancement		- Infiltrating growth - Hypervascularized - Hard capsule - Middle soft and yellowish
Osteoid osteoma (*n*=1; 0.75%)		
- Focal lytic lesion (“nidus”) within surrounding osteosclerotic reaction - MRI: enhancement of the nidus		- Macroscopically no tumor visible
Osteoma (*n*=16; 12%) Fig. 2f		
- Homogeneous bone-dense structure with partly cancellous partly hypersclerotic swelling - Exostosis of the tabula externa	Skeletal scintigraphy: - Focal uptake	- Solid bone swelling - Macroscopically intact bone structure - Soft borders around the bone
Secondary neoplasia		
Metastasis (*n*=19; 14%) Fig. 3a–b		
- Most common malignant bone lesion - In patients > 40 years, metastasis should always be considered in the case of a lytic lesion - Variable morphology: lytic, plastic, and mixed lesions - Variable contrast enhancement - Possible necrosis or hemorrhages - Dura infiltration	Skeletal scintigraphy: - Uptake PET: - Metabolically active	- Infiltrating growth - Lytic bone lesion - Tough and hard lesions - Bone thickening and very soft - Soft cancellous bone - Hypervascularized
Plasmacytoma (*n*=1; 0.75%) Fig. 3d		
- Uni- (plasmacytoma) or multifocal (multiple myeloma) lytic lesion - Numerous, well-circumscribed lytic lesions (“raindrop skull”)	X-ray: - Numerous lytic bone lesions	- Grown through the bone - Fibrotic tissue
Spindle cell melanoma (*n*=1; 0.75%) Fig. 3c		
- No specific imaging findings in case of bone involvement - The isolated case showed a tumorous mass of the scalp with transosseous and broad-based dural infiltration		- Reddened skin - Infiltrating growth
Squamous cell carcinoma (*n*=2; 1.5%)		
- Soft tissue process with perifocal osseous erosions - Bone destruction from the outside in, starting from the tabula externa - MRI: no specific findings		
Tumor-like lesions		
Aneurysmal bone cyst (*n*=1; 0.75%)		
- Very rarely in the calvaria - Typically age under 30 years - Well-circumscribed lytic lesion - MRI: multiple fluid levels with different signal behavior depending on the protein content		- Capsule strongly ingrown with the dura
Benign bone tissue with lytic lesions (*n*=1; 0.75%)		
- No specific imaging findings - The isolated case showed a circumscribed lytic lesion in the diploe without erosion of the tabula interna and externa		- Grayish-colored cancellous bone
CNS-Aspergillosis (*n*=1; 0.75%)		
- No specific imaging findings - Perifocal lytic bone lesion and infiltration of the adjacent tissue		- Altered, epidural, grayish tissue
Connective tissue (with foreign body granuloma) (*n*=3; 2%)		
- No specific imaging findings - The individual cases showed a lobulated, well-circumscribed lytic lesion with hyperintense signal in T2 and mixed signal in T1.		- Tough capsule - Dermoid-typical content - Connective tissue in the area of the lytic lesion - Dura thinned - Fat tissue
Fibrous dysplasia (*n*=6; 4%) Fig. 4c–e		
- Ground-glass opacities, homogeneously sclerotic - Thickened diploe - Mostly more than 1 bone affected (most often maxilla, orbit, frontal, ethmoid, and sphenoid bone) - MRI not indicated for primary diagnostic - Often not distinguishable from Paget’s disease. In fibrous dysplasia the tabula externa is more often affected	Skeletal scintigraphy: - Moderate focal uptake	- Hypovascularized - Grayish mass - Bone soft, hypertrophied, and reddish-glassy colored - No cancellous bone visible
Gorham-Stout disease (*n*=1; 0.75%)		
- Extremely rare - Nonspecific lytic bone lesion		- Very soft bone - Almost no cancellous bone
Histiocytosis (*n*=20; 15%) Fig. 4a–b		
- Well-circumscribed lytic bone lesion. Calvaria is most frequently affected (frontal > parietal > temporal > occipital bone), most rarely also mastoid, mandible, and orbit. Tabula interna more affected than tabula externa. - No periosteum reaction, possible perifocal soft tissue involvement - T1 iso-/hypointense; T2 hyperintense - Homogeneous contrast enhancement	Skeletal scintigraphy: - Uptake, moderately metabolically active	- Lytic bone lesion - Soft consistency - Grayish-glassy - Hypovascularized - Infiltrates the galea and periosteum
Paget’s disease (*n*=1; 0.75%)		
- Either well-circumscribed bone defects or diffuse sclerotic, ground-glass opacities of the bone - Thickened diploe - The calvaria is most frequently affected, in isolated cases also the skull base - MRI not indicated for primary diagnostic		- Manifest thickened cancellous bone
Reactive bone remodeling zone (*n*=1; 0.75%)		
- No specific imaging findings - The isolated case showed focal bone thinning with lobulated lytic lesion involving the diploe and the tabula interna and externa.		- Lytic bone lesion
Spindle cell tissue with abundant collagen connective tissue (*n*=1; 0.75%)		
- No specific imaging findings - The isolated case showed a central lytic lesion with sclerosis around the border		- Tough yellowish tissue
Venous ectasia (*n*=1; 0.75%)		
- Like hemangioma - Well-circumscribed lytic lesion - Thin peripheral sclerotic border - No destruction of tabula interna and externa - Expansion of the bone trabeculae in the diploe - MRI: in T2 heterogeneous hyperintense signal, in T1 with contrast agent diffuse enhancement with time-delayed filling	Sonography: - Liquid-filled cavity - Intracranial connection - CFD: minimal flow	- Bone thinned

A complete resection has been achieved in 80% of cases (*n*=107). A partial removal was performed in 14% (*n*=18), a biopsy in 6% (*n*=8) of cases. Intraoperative complications have only occurred in a few cases: intraoperative bleeding in 10 patients (7%; histologically metastasis, meningiomas, epidermal cyst, and melanoma), of which 3 are due to a sinus injury (2%). Accidental opening of the dura mater occurred in 7 patients (5%).

Fifty-eight percent of the lesions (*n*=77) were limited to the bone. The remaining lesions (*n*=56; 42%) have shown an infiltrating growth in the adjacent tissue, especially in the dura in 32% of cases (*n*=43). Infiltration up to arachnoid mater has been shown in 1.5% of cases (*n*=2), up to the brain in 12% of cases (*n*=16). Extracranially, infiltration to the skin has been shown in 10% of cases (*n*=13), to the subcutaneous tissue in 4% of cases (*n*=5), to the muscles in 1.5% of cases (*n*=2), and to the galea in 9% of cases (*n*=12). The described macroscopic peculiarities of the respective lesions are listed in Table 1.

### Postoperative course

The final histopathological results are listed in Table 1. Postoperative complications have occurred in <5% of cases: cerebrospinal fluid fistula in 4% (*n*=5), temporary neurological deficits in 3% (*n*=4), sinus thrombosis in 2% (*n*=3), wound healing disorders in 1.5% (*n*=2), and an epidural hematoma in 0.75% of cases (*n*=1). In the documented follow-ups (FU) (3–120 months), 10% of patients (*n*=14) still reported pain or pressure at the site of surgery, 4% (*n*=5) paresthesia, and another 4% (*n*=5) still had a slight swelling in the surgical area. No permanent neurological disorders or deaths occurred.

In patients with primary neoplasia (55%; *n*=73; Table 1), a complete resection was performed in 44% (*n*=59) of cases without developing a recurrence (FU 3–120 months, mean 32, SD 31). Partial resected meningiomas have a stable residual result in 6% (*n*=8) of cases (FU 3–57 months, mean 25, SD 19.4). In 4.5% of cases (*n*=6; 5 meningiomas and 1 epidermoid), there was a recurrence within 12–72 months (mean 29, SD 24). The meningiomas needed an adjuvant therapy, and the recurrent epidermoid has been resected again.

Secondary neoplasia (18%; *n*=24; Table 1) has been completely resected in 7.5% (*n*=10) of cases (FU 3–9 months, mean 5, SD 2.5). Adjuvant therapy has occurred in 12% (*n*=16) of cases, of which 1.5% of cases remained stable (*n*=2, histologically metastases, FU 36 months), 0.75% decreasing (*n*=1, histologically plasmacytoma, FU 28 months), and 5% have shown recurrent growth (*n*=7, histologically metastases, squamous cell carcinoma, and melanoma; FU 3–84 months, mean 34, SD 31). Four percent (*n*=5) have been operatively resected again, 1.5% of which (*n*=2) were only on the scalp (squamous cell carcinoma).

Tumor-like lesions (27%; *n*=36; Table 1) have been completely resected in 19.5% of cases (*n*=26) without recurrence (FU 3–114 months, mean 43, SD 35). In 4.5% of cases (*n*=6, histologically fibrous dysplasia, Gorham-Stout disease, Paget’s disease, histiocytosis; FU 4–95 months, mean 40, SD 39), a further treatment was performed with a stable course. In this group, recurrence has not been observed.

Eight patients (6%) got lost from FU.

## Discussion

The series of calvarial lesions presented here shows a variety of different entities (Table 1). Their incidence correlates with the cases already published [8, 9, 14, 17]. Depending on the author, the lesions that primarily originate from the bone are divided into three or four subcategories: primary neoplasia (in individual papers already divided into good and malignant primary neoplasia), secondary neoplasia, and tumor-like lesions [17, 22].

**Fig. 2:**
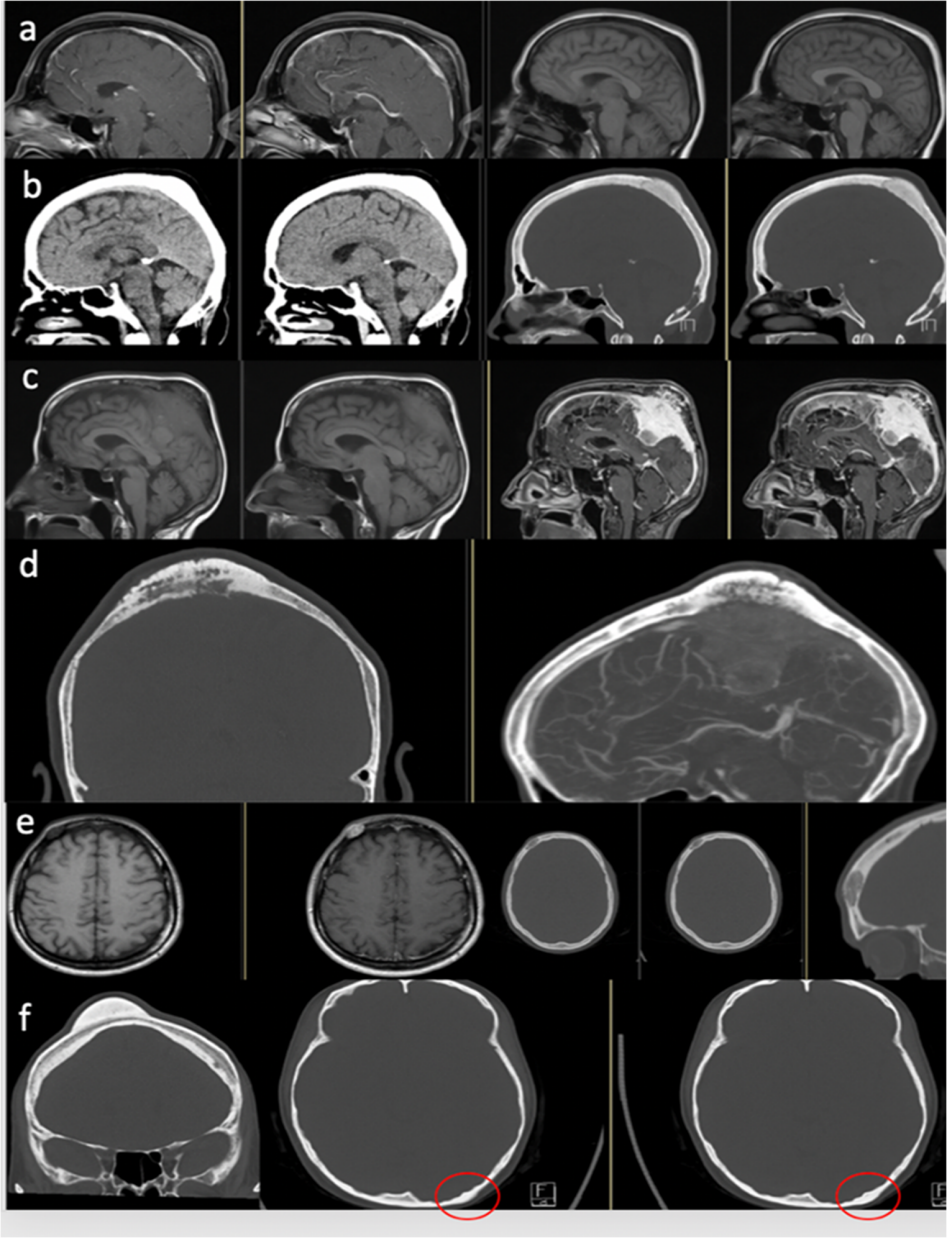
Primary neoplasia. **a** + **b** Intraosseous meningioma WHO I. **c** + **b** Meningioma WHO Grad III with infiltrative growth. **e** Intraosseous hemangioma. **f** Osteoma

Primary osseous neoplasia come from the bone and are distinguished into benign and malignant lesions. Primary malignant lesions such as chordomas or sarcomas are not represented in our series, since they are frequently located at the skull base. Because a complete resection is not always possible, it is advisable to perform at least one biopsy or extended removal to ensure the diagnosis and initiate adjuvant treatment [9, 10]. The most common benign lesions include meningiomas (Fig. 2a–d), osteomas (Fig. 2f), and intraosseous hemangiomas (Fig. 2e). Osteomas (Fig. 2f) grow slowly and are located on the tabula externa. The main complaints are the cosmetically disturbing swelling, less than the pain. If they are localized at the orbita, diplopia can occur [1]. Imaging shows a densely compact bone growth. No further treatment is necessary after a complete resection and there is an extremely low risk of recurrence [8, 9, 14]. Intraosseous hemangiomas (Fig. 2e) are described in the literature more in women. As long as they are limited to the bone, they are often an asymptomatic random result [15, 16, 18]. Further growth can cause painful swelling. A typical “sunburst” phenomenon is shown in the CT [18]. In the MRI, a variable arterio-venous contrast enhancement is described [8]. Complete resection down to healthy bone is recommended to minimize the recurrence risk [8, 9, 14, 15, 17, 18]. Meningiomas (Fig. 2a–d) are among the most common benign intracranial tumors. However, about 2% of meningiomas grow ectopic in the bone and infiltrate the adjacent tissue only secondarily [5, 25]. Typical imaging findings are calcifications, homogeneous contrast enhancement, edema, and a dural tail [8, 9, 14]. Our series includes different WHO grade of meningiomas, which also have significant different infiltrating growth. Adequate preoperative imaging is essential to minimize the intraoperative and postoperative risk for the patient. A complete resection is also recommended for meningiomas. If this is not possible, appropriate adjuvant therapy should be evaluated depending on the histology [5].

**Fig. 3:**
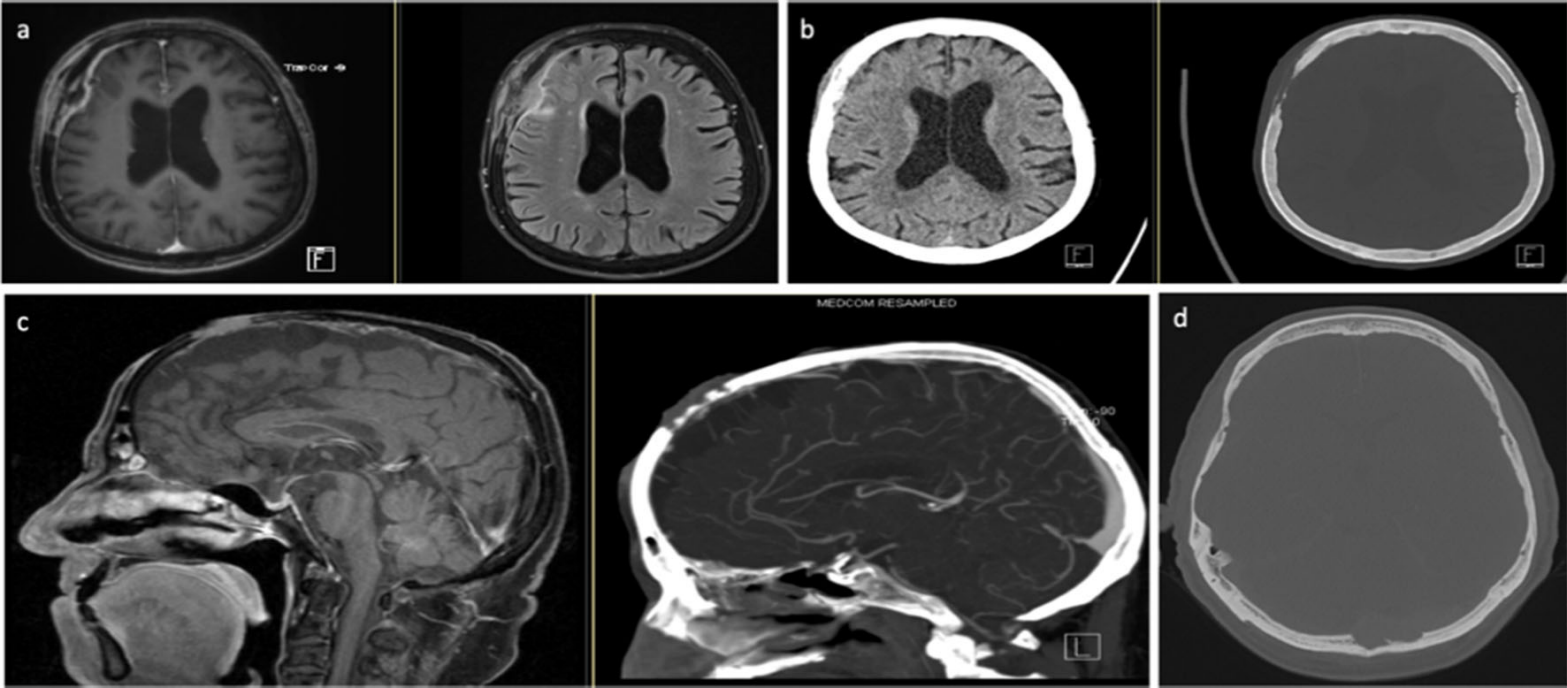
Secondary neoplasia. **a** + **b** Metastasis. **c** Malignant melanoma with infiltrative growth and bone destruction. **d** Osteolytic lesion in plasmacytoma

The most common entities of secondary neoplasia are metastasis (Fig. 3a–b) of different primary carcinomas. In most cases, they are asymptomatic and stand out as random findings in the regular staging examinations. Patients can develop pain when the periost is infiltrated. By intracranial growth, the patients can also develop neurological deficits. Typical of the imaging are irregular osteolysis with remodeling of the bone, a variable contrast enhancement, and signs of a co-reaction of the surrounding tissue, for example, a perifocal edema [8, 9, 14, 22]. A complete resection must be sought, and subsequently, an adjuvant treatment should be started depending on the primary carcinoma [13, 17, 22]. The osteoclastic defects of a plasmacytoma (Fig. 3d) also fall into the category of secondary neoplasia. Typical are pure osteolysis without remodeling of the bone [8]. Surgical resection with cover of the bone defect is recommended. In the event of systemic infestation, further treatment of the disease is necessary. In the scenario of a solitary manifestation and complete surgical resection, close-meshed follow-ups are necessary. In this case, further treatment is controversially discussed in the literature [3, 6, 11, 19]. In the case of a histologically secured secondary neoplasia, a staging examination with whole body CT scan should be performed in order to evaluate the spread of the disease and detect other possible bone lesions.

**Fig. 4:**
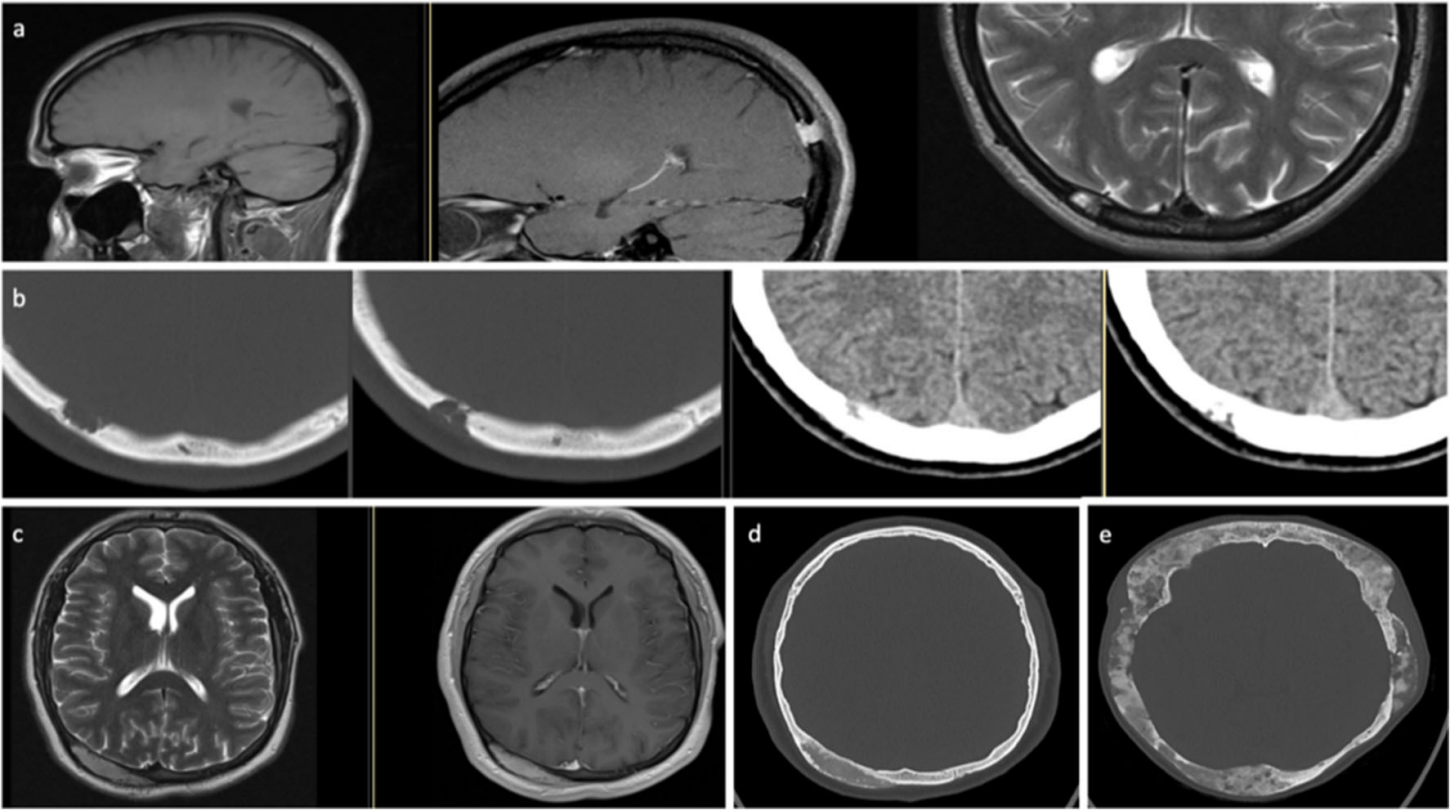
Tumor-like lesions. **a** + **b** Histocytosis. **c** + **d** Monostotic fibrous dysplasia. **e** Polyostotic fibrous dysplasia

In the group of tumor-like lesions, there are, in particular, histiocytosis (Fig. 4a–b), especially eosinophilic granuloma, and metabolic disorders, such as fibrous dysplasia (Fig. 4c–e). Histiocytosis (Fig. 4a–b) is a group of diseases with an increased proliferation of macrophages. Typical imaging findings are osteolysis with singular bone fragments within the lesion, as a sign of irregular bone destruction. MRI shows a homogeneous contrast enhancement limited to the bone without a co-reaction of the dura or perifocal edema (Table 1) [8, 9, 20]. The diagnosis can only be secured histologically. After this, a staging should been performed. If the lesion is isolated at the skull, no further therapy is necessary, but for multifocal manifestations, chemotherapy and radiotherapy are recommended [2, 20]. Another known bone malformation is fibrous dysplasia (Fig. 4c–e), which is due to overactivity of osteoclasts and leads to disturbed bone differentiation [7]. The malformation can affect all bones of the body, including the skull. Patients often complain of severe pain and have swelling or fractures. A distinction is made between the monostotic form (Fig. 4c–d), which can be treated by surgical resection and covering the defect [4], from the polyostotic form (Fig. 4e), in which symptomatic treatment with analgesics and bisphosphonates takes place. In the polyostotic form, only partial removal makes sense if important structures are compressed and patients develop complaints [7]. A typical finding in the CT-scan is ground-glass opacities [4, 8, 17].

In our series, secondary, the bone-infiltrating lesions also occurred, such as squamous cell carcinoma and malignant melanoma (Fig. 3c). These, too, have an osteolytic growth; depending on the stage of the disease, the infiltration can range from the skin to intracranial. Surgical resection and cover of the defect with subsequent systemic adjuvant therapy must be planned [21, 24].

Based on the imaging, suspicions of the underlying lesion can be obtained (Table 1 and Figs. 2, 3, and 4). The limits of a lesion can suggest conclusions about the speed of growth: smooth edges and a homogeneous internal structure mark more of a benign, slowly growing process; irregular edges with inhomogeneous internal structure point, rather, to a malignant, fast-growing process. Also, slowly growing lesions are usually limited to the bone, while fast-growing show an infiltrating growth in adjacent tissue. With the administration of a contrast agent, further conclusions can be drawn about the dignity of the lesion. While reactive or scarring changes in the dura have a linear, homogeneous contrast enhancement, tumor infiltration is often characterized by irregular, inhomogeneous contrast enhancement. If the dura looks distant from the bone and, thus, the epidural space is visible, a dura infiltration is unlikely [8, 9, 12, 14, 17].

The management of patients with calvarial lesions should be interdisciplinary, as with other oncological diseases. The general practitioner is usually the first contact for the patient, as the lesions are often random results and there are no complaints. To draw conclusions about the possible entity of the lesion, a targeted image is required [8, 9, 12, 14, 17]. The suspected diagnoses can be limited and the further procedure planned. In any case, a surgical extraction of a tissue sample is necessary to ensure the diagnosis. If properly planned, the operation is a low-risk procedure without significant morbidity and mortality [12, 17, 22]. Important imaging aspects are, in particular, the extension of possible infiltration and the course of the large blood vessels, especially intracranially of the sinus. A precisely planned surgical procedure minimized the risks of bleedings and the intraoperative blood loss. Therefore, we recommend the implementation of a CT and MRI with and without contrast agent. In case of lesions near larger blood vessels, a CT or MR angiography or phlebography should also occur. Some lesions can have a similar extension on the bone, but differ significantly in their intracranial spreading (Fig. 2a–b vs c–d). The medical history, clinical complaints, and differential diagnoses must be taken into account together to evaluate the urgency of histological examination. While an underlying carcinoma requires a timely confirmation of a possible metastasis to initiate a targeted adjuvant treatment, in the case of a purely intraosseous lesion with a homogeneous internal structure and without any co-reaction of the adjacent tissue and without any complaints, an initial follow-up observation is also acceptable and surgical care is not urgent. If an operation takes place, a complete resection with cover of the defect must be planned.

## Data Availability

All data are included in the manuscript.
